# Vacuolated PAS-Positive Lymphocytes on Blood Smear: An Easy Screening Tool and a Possible Biomarker for Monitoring Therapeutic Responses in Late Onset Pompe Disease (LOPD)

**DOI:** 10.3389/fneur.2018.00880

**Published:** 2018-10-22

**Authors:** Daniela Parisi, Olimpia Musumeci, Stefania Mondello, Teresa Brizzi, Rosaria Oteri, Alba Migliorato, Annamaria Ciranni, Tiziana E. Mongini, Carmelo Rodolico, Giuseppe Vita, Antonio Toscano

**Affiliations:** ^1^Department of Clinical and Experimental Medicine, University of Messina, Messina, Italy; ^2^Department of Biomedical and Dental Sciences and Morphofunctional Imaging, University of Messina, Messina, Italy; ^3^DIBIMIS University of Palermo, Palermo, Italy; ^4^Department of Neurosciences Rita Levi Montalcini, University of Turin, Turin, Italy

**Keywords:** PAS-positive lymphocytes, blood smear, LOPD screening test, therapeutic monitoring, Pompe disease

## Abstract

**Background:** Primary aim was to investigate the diagnostic value of PAS-positive vacuolated lymphocytes on blood smear in Late Onset Pompe Disease (LOPD) patients and, secondly, to evaluate its potential utility in monitoring treatment effects.

**Methods:** We examined blood smear of 26 LOPD patients. We evaluated 10 treated and 16 untreated LOPD patients. Among the latter group, 7 patients later initiated ERT and were tested again 6 months after start. Blood smear was also sampled from 82 controls and 19 patients with other muscle glycogenoses (MGSDs). PAS staining was used to evaluate: (1) presence of lymphocytes with glycogen-filled vacuoles, (2) quantification of vacuolated lymphocytes.

**Results:** We found that PAS-positive lymphocytes were significantly higher in LOPD patients than in controls or other MGSDs (*p* < 0.05 and *p* < 0.001, respectively). ROC curve for discriminating between untreated LOPD patients and controls yielded an AUC of 1.00 (95%CI 1.00–1.00; *p* < 0.0001). PAS-positive lymphocyte cutoff level of >10 yielded sensitivity of 100% (95%CI 78–100%), specificity of 100% (95%CI 96–100%), and positive predictive value of 100%. Patients studied before and after ERT showed a dramatic decrease of PAS-positive vacuolated lymphocytes number (*p* = 0.016). In other MGSDs, PAS-positive lymphocytes were significantly lower that untreated LOPD patients but higher than controls.

**Conclusions:** Our data suggest that the Blood Smear Examination (BSE) for PAS-positive lymphocytes quantification could be used as a simple and sensitive test for a quick screening of suspected Pompe disease. The quantification of vacuolated lymphocytes appears to be also a valuable tool for monitoring the efficacy of treatment in LOPD patients.

## Introduction

Pompe disease (glycogen storage disease type II, OMIM#232300) is a rare autosomal recessive lysosomal storage disorder caused by deficiency of acid alpha-glucosidase (GAA), a lysosomal enzyme that is responsible for the cleavage of the α-1,4- and α-1,6-glycosidic bonds of glycogen to glucose (1, 2).

GAA deficiency leads to the accumulation of glycogen in the lysosomes of several tissues, demonstrating a multisystemic disorder although cardiac and skeletal muscles involvement remains more prominent (3, 4).

Two different clinical forms are conventionally described: a severe infantile form (IOPD) characterized by muscular hypotonia, hypertrophic cardiomyopathy and respiratory failure, and a more heterogeneous late onset form (LOPD) with a predominant progressive proximal, axial and respiratory muscle weakness (5–7).

In LOPD, initial clinical manifestations as muscle weakness, exercise intolerance, myalgia, or even isolated hyperCKemia appear often unspecific and may mimic a large variety of other muscle disorders as limb-girdle muscular dystrophies (LGMD), congenital, metabolic or inflammatory myopathies (8–11).

^1^According to the recent European Pompe Consortium (EPOC) recommendations for a correct Pompe disease diagnosis, a rapid and appropriate Dried Blood Spot (DBS) test may detect reduced GAA activity (12–14). This method may allow a fast screening of LOPD high-risk populations, so providing an addressing role in the diagnostic algorithm (14–16). In case of positive result it is necessary to perform a second biochemical confirmatory test on a different tissue (leucocytes, fibroblasts, or skeletal muscle) and/or the molecular genetic analysis (12).

However, muscle biopsy remains an important tool in the evaluation of muscle disorders; in most of Pompe disease cases, the morphological study shows a pattern of vacuolar myopathy with glycogen storage but sometimes it can result unspecific (17).

Since 2006, Enzyme Replacement Therapy (ERT) with recombinant human α-glucosidase (rGAA) became available. Early initiation of ERT in symptomatic patients seems to be essential to limit the progressive muscle damage, emphasizing the need for an early diagnosis (12, 18–20).

Abnormal cytoplasmic vacuolation of lymphocytes, identifiable on blood smear examination (BSE), has been proposed as a possible screening tool in Pompe patients (21–23).

The aim of the present study was to primarily investigate the diagnostic value of BSE of vacuolated lymphocytes in a cohort of LOPD patients compared to sex- and age-matched healthy individuals and to other patients with different muscle glycogenoses (MGSDs). Further, we evaluated the possibility of using BSE of vacuolated lymphocytes as a biomarker for monitoring and assessing treatment effects in LOPD.

## Methods

### Study population

The study was approved by the local ethics committee (University Policlinic of Messina). The research was conducted according to the revised Declaration of Helsinki (1998) and all participants provided written informed consent prior to participation in the study.

Between April 2015 and March 2017, we examined blood smears of 26 patients defined diagnosis of LOPD, followed at our Neuromuscular Unit. Subjects were 15 males and 11 females, aged from 3 to 78 years. In all patients, the diagnosis of Pompe disease was confirmed by GAA activity assay on skeletal muscle and genetic analysis as recently suggested by EPOC recommendations (12).

When the study began, a blood sample was obtained from 10 treated and 16 untreated LOPD patients. During the study course, 7 out of the 16 untreated patients initiated ERT and, to monitor the effects of therapy on lymphocytes vacuolations, patients were tested again 6 months after ERT start.

Control values were obtained from 82 age-matched healthy individuals —40 M and 42 F—aged from 12 to 90 years.

We also collected blood smears from 19 patients affected by other muscle glycogenoses: 9 with glycogenosis type V (GSD V), 2 with GSD VII, 5 with GSD III, 1 with GSD X, 1 with GSD XIII and another one with GSD 0.

### Assessment of vacuolated peripheral blood lymphocytes

A blood sample was taken and two blood films were prepared for each subject. Using routine staining procedures for light microscopy, blood smears were stained by Periodic Acid-Schiff (PAS) stain to evaluate the lymphocytes with glycogen-filled vacuoles (23, 24). Laboratory staff performing sample analysis was blinded to clinical information.

The number of vacuolated PAS positive lymphocytes per 100 lymphocytes (percentage of PAS-positive lymphocytes) was counted.

**Figure 1:**
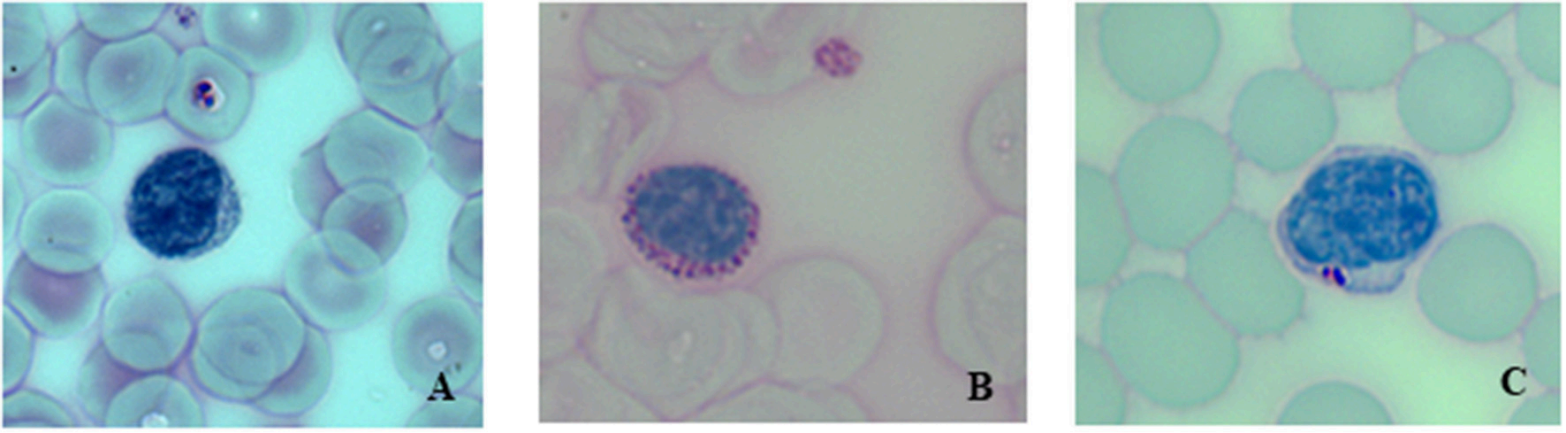
Periodic acid-Schiff (PAS)-stained blood smear at 40 X magnification. **(A)** Healthy control, **(B)** Untreated LOPD patient showing a lymphocyte with a larger number of PAS-positive inclusions, **(C)** Same LOPD patient after 6 months of ERT.

Figure 1 shows PAS-stained blood smear of a healthy individual (A), of an untreated LOPD (B) and of the same LOPD patient after ERT (C).

### Statistical analyses

Data were assessed for equality of variance and distribution. Descriptive statistics with means and median, as appropriate, and proportions were used to describe continuous and categorical variables. The association between categorical variables and population group was evaluated using the chi-square test. Because of the skewed distribution, Mann–Whitney U test was used for 2 continuous group comparisons and or the Kruskal–Wallis test for 3 or more continuous group comparisons. To compare pre and post-ERT PAS-positive lymphocytes values, we used the Wilcoxon paired rank test. Receiver operating characteristic (ROC) curves were created to explore the ability of PAS-positive lymphocytes to distinguish between LOPD patients and controls. Estimates of the area under the curves were obtained (area under the curve = 0.5 indicates no discrimination and an area under the curve = 1.0 indicates a perfect diagnostic test). PAS-positive lymphocyte cut point was selected to maximize the sensitivity and specificity. Classification performance was assessed by sensitivity, specificity, and positive predictive values with 95% CIs. All statistical tests were two-sided and a *p-*value < 0.05 was considered statistically significant. Statistical analysis was carried out using SAS software package version 9.4 (SAS Institute Inc., Cary, NC) and R software (www.r-project.org; version 3.3.3).

## Results

We studied a total of 26 LOPD patients, 82 healthy individuals and 19 patients with other MGSD, enrolled from April 2015 until March 2017. The demographic and clinical characteristics of patients and controls are shown in Table 1.

**Table 1:** Summary of demographic and clinical data of LOPD cases as well as healthy individuals included in this study.

	**Healthy individuals (*n* = 82)**	**Patients with LOPD (*n* = 26)**	***p*-Value^a^**
**Age, years**, median (IQR) Range	53 (34–71) (12–90)	50 (37–55) (3–78)	0.09
**Male**, *n* (%)	40 (49%)	15 (57.69%)	0.63
**Age at Onset years**, median (IQR)	NA	32.5 (20–40)	
**Age at Diagnosis, years**, median (IQR)	NA	43 (34–51)	
**CLINICAL PRESENTATION**, ***n*** **(%)**			
– Isolated hyperCKemia	NA	7 (26.92%)	
– LGMW	NA	19 (73.08%)
**ERT**, ***n*** **(%)**			
– Yes	NA	10 (38.46%)	
– No	NA	16 (61.54.08%)	

a *Mann–Whitney U test for continuous variables, cross-tabulations and 2-test for categorical variables*.

*NA, not applicable*.

In this LOPD cohort, 7 patients only showed presymptomatic hyperCKemia whereas 19 manifested with axial and limb girdle muscle weakness. Muscle biopsy, performed in 24 out of 26 LOPD patients, showed a variable amount of fibers with glycogen-filled vacuoles in 92% of patients.

The 19 patients with other MGSDs (10 M and 9 F) aged from 6 to 58 years. 9 GSD V and 2 GSD VII patients presented hyperCKemia, exercise intolerance and myoglobinuria without muscle weakness. GSD X and XIII patients complained of exercise intolerance, myalgia and contractures and rhabdomyolysis episodes. GSD III patients presented axial and limb-girdle muscle weakness. GSD 0 patient presented dysmorphic features with short stature, long face and low ears, exercise intolerance, and respiratory failure.

In all Pompe patients, we found, on a blood smear, a high percentage of vacuolated PAS-positive lymphocytes ranging from 10 to 57% in untreated and from 2 to 28% in treated patients that resulted significantly different than controls (*p* < 0.01 and *p* < 0.001, respectively) (Table 2).

**Table 2:** Number of vacuolated-PAS positive lymphocytes in the study cohort of LOPD patients, in other MGSDs and in healthy individuals.

	**Healthy individuals (*n* = 82)**	**Untreated LOPD patients (*n* = 16)**	**Treated LOPD patients (*n* = 10)**	**Other MGSDs (*n* = 19)**	***P*-Value^a^**
Number of vacuolated PAS positive lymphocytes (IQR)	1 (0–3)	37 (18–45)	9 (2–17)	3(1–9)	< 0.0001
Range	(0–9)	(10–57)	(2–28)	(0–17)	

a *Kruskal-Wallis test for continuous variables, IQR, interquartile range*.

GAA activity showed a strong negative correlation with PAS-positive lymphocytes in both treated and untreated LOPD patients (*r* = −0.75, *p* = 0.01, and *r* = −0.60, *p* = 0.02, respectively). On the other hand, PAS-positive lymphocytes weakly correlated with age in controls (*r* = −0.27, *p* = 0.015) and approached the significance in treated LOPD patients (*r* = −0.58, *p* = 0.07). Conversely, PAS-positive lymphocytes were not associated to clinical phenotype (*p* = 0.53) or any other correlation. No correlation with genotype was found.

**Figure 2:**
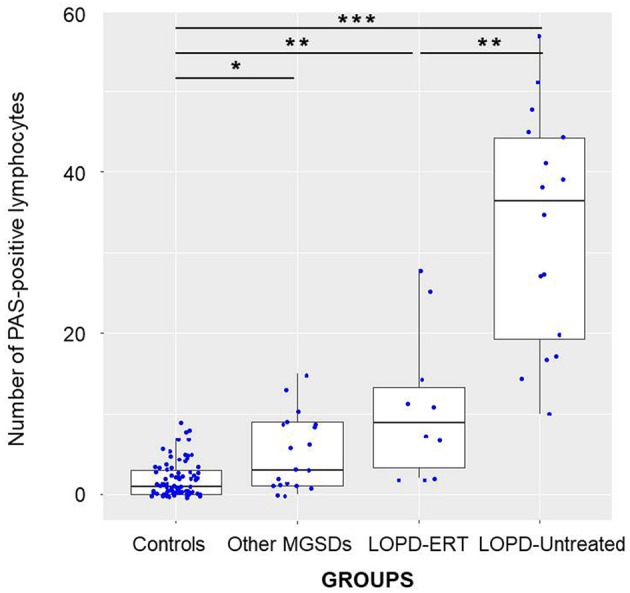
A comparison of the number of vacuolated lymphocytes in patients with LOPD dichotomized into untreated and ERT-treated vs. controls and other MGSD. The black horizontal line in each box represents the median, with the boxes representing the interquartile range. Significant differences are indicated with *p < 0.05, **p < 0.01, or ***p < 0.001.

PAS-positive lymphocytes in patients with MGSD, although they were significantly higher than in controls (*p* < 0.05), appeared to be statistically lower than untreated LOPD patients (*p* < 0.01), and tended to be lower compared to treated patients (Figure 2). Intriguingly, in GSD III cases, the presence of vacuolated lymphocytes was higher than in the other MGSD making the MGSD mean values in the lower level of the untreated LOPD patients.

**Figure 3:**
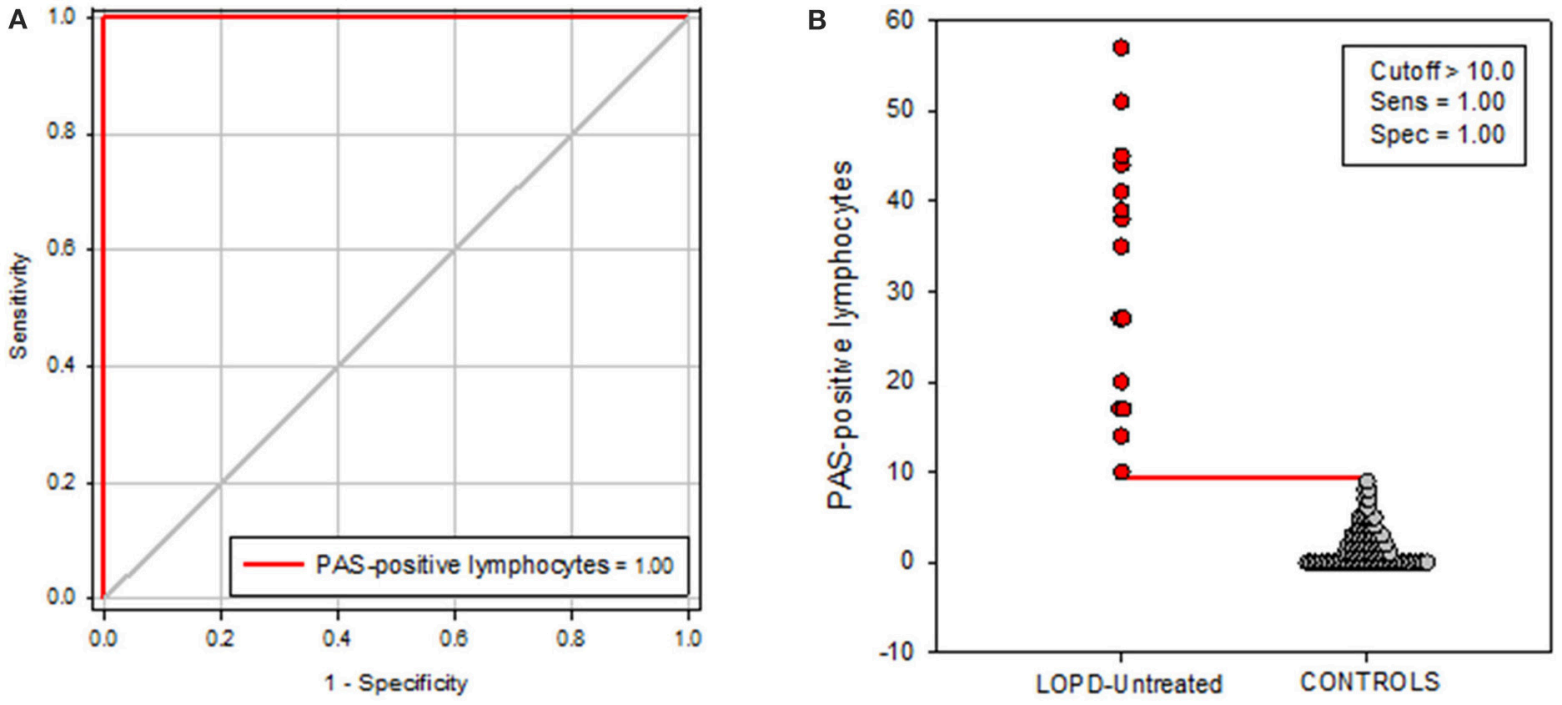
**(A)** Receiver operating characteristic (ROC) curve for distinguishing untreated LOPD patients vs. controls. The area under the receiver operating characteristic curve (AUC) demonstrates that the levels of PAS-positive lymphocytes assessed in serum are able to discriminate between untreated LOPD patients and controls, with an AUC of 1.00 (95% CI 1.00 to 1.00). **(B)** Levels of PAS-positive lymphocytes in untreated LOPD patients and in controls. The red horizontal line represents the optimal cutoff value for distinguishing between untreated LOPD patients and controls.

ROC curve for discriminating between untreated LOPD patients and controls yielded an area under the curve (AUC) of 1.00 (95% CI 1.00–1.00; *p* < 0.0001) (Figure 3).

Classification performance at a PAS-positive lymphocyte cutoff level of >10 yielded a sensitivity of 100% (95% CI 78 to 100%), a specificity of 100% (95% CI 96 to 100%), and a positive predictive value of 100%.

**Figure 4:**
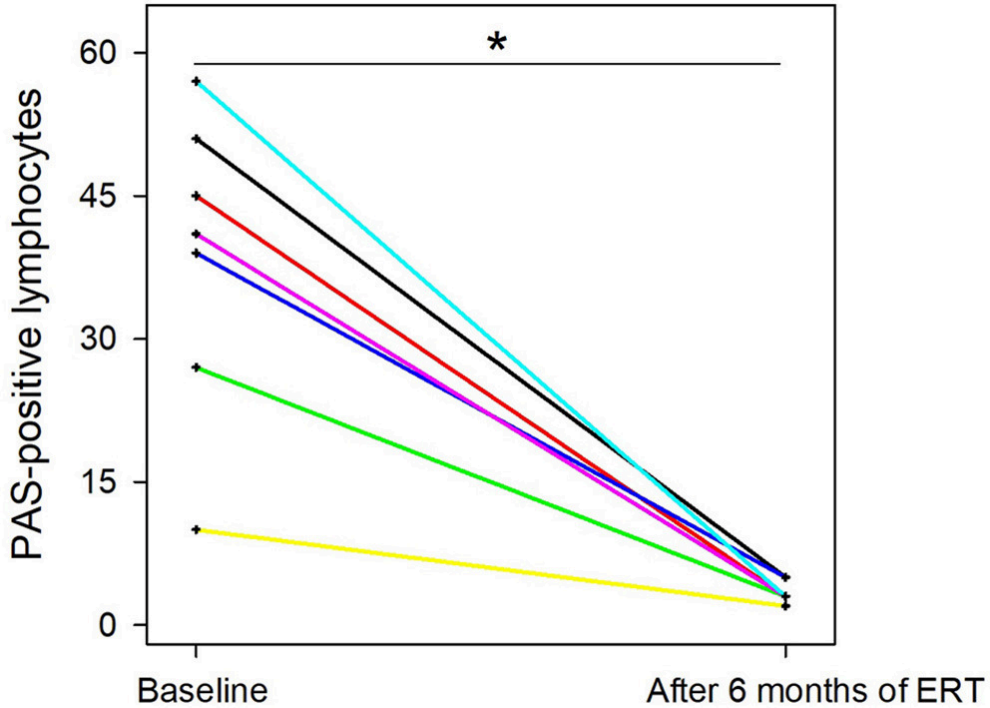
Trends over time in the number of vacuolated lymphocytes *P = 0.016. Individual patient values are showed.

We compared PAS- positive lymphocytes in blood samples, obtained from 7 patients before starting ERT and after 6 month of treatment. Patients showed a dramatic decrease in the number of PAS-positive lymphocytes after 6 months on ERT compared to baseline values (*P* = 0.016) (Figure 4).

## Discussion

Since the availability of ERT, as first line treatment for patients with Pompe disease, it became evident that an early diagnosis is crucial to achieve efficient therapeutic responses. The use of the DBS as a key screening method to identify patients with GAA deficiency has been proposed in several studies (14–16).

In Pompe disease, glycogen storage is present in different tissues including lymphocytes in the peripheral blood. It has been reported that on BSE, patients with Pompe disease may show vacuolated lymphocytes (21–23).

In 1977, von Bassewitz et al observed vacuoles in peripheral lymphocytes by light microscopy and, using electron microscopy, detected glycogen-filled lysosomes in 5 IOPD cases (21). In the following years, vacuolated lymphocytes have been described on blood smear also in other storage and metabolic disorders as Batten's disease (neuronal ceroid lipofuscinosis), Salla disease, β galactosidase deficiency, mucopolysaccharidoses, Niemann-Pick disease, fucosidosis, mannosidosis, and Wolman's disease. In 2005, a retrospective review of 2.550 blood films of patients with a clinical history suggestive of metabolic diseases, identified vacuolated lymphocytes in 156 cases, 23% were recognized as Pompe disease (15 IOPD and 8% LOPD) PAS staining was performed to better characterize glycogen storage in the lymphocytes vacuoles (22). In 2010, Hagemans et al collected peripheral blood films from patients with Pompe disease and controls showing that PAS-positive lymphocytes were more common in Pompe disease compared to controls and suggesting their possible role as diagnostic screening procedure (23). More recently Pascarella et al. suggested that quantification of PAS-positive lymphocytes in blood films is useful to identify autophagic vacuolar myopathies and should be routinely used for Pompe disease diagnosis (25).

In this study, we investigated the presence of glycogen-filled vacuoles in peripheral blood lymphocytes of LOPD patients to evaluate its use as screening test as well as surrogate biomarker to monitor therapeutic efficacy. Our data confirmed that PAS staining is a reliable marker of glycogen accumulation in LOPD patients lymphocytes. Comparing the number of PAS-positive lymphocytes of all 26 LOPD patients vs. controls or others MSGDs patients, we found that they were significantly higher in LOPD (Figure 2), proving that this method is quite specific to detect Pompe disease patients. A strong correlation was found between presence of vacuolated lymphocytes and GAA residual activity but not with other clinical parameters as age, disease duration or phenotype.

On the other hand, considering treated and untreated LOPD patients, we found that PAS-positive lymphocytes were significantly higher in untreated than in treated patients (*p* < 0.01). Diagnostic accuracy of the PAS-positive lymphocytes in blood was quite impressive showing an AUC of 100%. With a cutoff value of 10 as a percentage of PAS positive lymphocytes, a sensitivity of 100% and a specificity of 100% were reached (Figure 3). Thus, these results indicate that this test may play a role as valid and reliable indicator of LOPD.

It is worthwhile to outline that, in 7 LOPD patients, the percentage of PAS-positive lymphocytes, counted in blood smears before ERT and 6 months after, was significantly lower after treatment (Figure 4). Although number of samples is quite limited, the latter finding could suggest that the percentage of the PAS-positive lymphocytes could be utilized as a surrogate biomarker to check therapeutic efficacy, even in future trials. Similarly in Fabry disease, another lysosomal disorder which share several commonalities with Pompe disease, GB3 and Lyso-GB3 in plasma and urine are considered as reliable biomarkers for staging the disease and monitor ERT response (26).

PAS-positive lymphocytes values of patients with other MGSDs were significantly reduced than in untreated LOPD patients. They also tended to be lower compared to treated LOPD patients (Figure 2), suggesting that BSE could be usefully applied as a screening tool in LOPD high-risk population, even in combination with DBS. Of course, it is worthwhile to outline that in the LOPD diagnostic algorithm, BSE as well as DBS results need to be confirmed by biochemical and genetic testing.

Being based on a simple histochemical test, BSE could be even easier to be applied in patients screening rather than DBS methods that require a fluorimetric or a tandem mass spectrometry, equipment that could be not universally available in the setting of diagnostic laboratories.

However, GSD III patients seem to have higher PAS-positive lymphocytes than other MGSDs; they appeared quite similar to the lowest Pompe disease untreated values in this cohort although GSD III clinical features are usually distinctive from Pompe disease (27). A possible explanation of a similar morphological appearance of vacuolated lymphocytes in GSD II and III, should take into account the fact that even debrancher enzyme is located in lymphocytes and its deficiency may lead to glycogen accumulation (28).

Our results have shown that residual GAA activity strongly correlated with PAS-positive lymphocytes in both treated and untreated LOPD patients. One limit of the study could be considered the relatively small sample size that precluded meaningful multivariate analyses.

## Conclusions

Our data suggest that quantification of PAS-positive lymphocytes in peripheral blood films could be used either as a simple screening method to support a diagnosis in patients with a suspected Pompe disease or also as surrogate biomarker for therapeutic management purposes.

## Compliance with ethics guidelines

All procedures followed were in accordance with the ethical standards of the responsible committee on human experimentation (institutional and national) and with the Helsinki Declaration of 1978. Informed consent was obtained from all patients for being included in the study.

## Author contributions

OM had full access to all the data in the study and takes responsibility for the integrity of the data and accuracy of data analysis. DP, OM, AT, TM, CR, GV contributed to study design. DP, OM, TB, RO, AC, AM, AT contributed to data collection. DP, OM, AT drafted the manuscript. OM, TB, CR, TM provided clinical information. SM performed statistical analysis. All authors read and approved the final manuscript.

### Conflict of interest statement

In the last 3 years, AT has received from Sanofi Genzyme some reimbursement for talking in teaching courses and because he also is member of Global Pompe Registry committee. OM received reimbursement for participation in invited lectures by Sanofi Genzyme. The remaining authors declare that the research was conducted in the absence of any commercial or financial relationships that could be construed as a potential conflict of interest.

## Footnotes

^1^Abbreviations: LOPD - Late onset Pompe disease; GSD glycogenoses - MGSD - muscle glycogenoses; LGMW - limb girdle muscle weakness; MGG - MayGrünwald/Giemsa; PAS - Periodic Acid-Schiff; GAA - acid alpha-glucosidase; DBS - dried bloodspot; BSE - Blood Smear Examination; GAA - acid alpha-glucosidase; IOPD – infantile onset Pompe disease; EPOC - European Pompe Consortium; ERT – Enzyme Replacement Therapy; ROC - Receiver operating characteristic; IQR - interquartile range; AUC - area under the curve.
